# A case of lymphocytic esophagitis in a woman with multiple allergies

**DOI:** 10.1186/s13223-021-00558-x

**Published:** 2021-06-07

**Authors:** O. Wojas, M. Żalikowska-Gardocka, E. Krzych-Fałta, B. Szczepankiewicz, P. Samel-Kowalik, B. Samoliński, A. Przybyłkowski

**Affiliations:** 1 Department of Prevention of Environmental Hazard and Allergology, Medical University of Warsaw, Warsaw, Poland; 2 Department of Gastroenterology and Internal Medicine, Medical University of Warsaw, Warsaw, Poland; 3 Department of Pathomorphology, Medical University of Warsaw, Warsaw, Poland

**Keywords:** Allergy, Dysphagia, Endoscopy, Eosinophilic esophagitis, Lymphocytic esophagitis, GERD

## Abstract

**Background:**

Lymphocytic esophagitis is a newly recognized entity of unknown origin. Dysphagia is defined as difficulty swallowing and represents a common symptom in the general population with a prevalence of approximately 20%. Chronic inflammation of the esophageal wall may manifest itself clinically and endoscopically, mimicking inflammation of another origin. However, little is known about the pathogenesis of the disease, as patients are seldom suspected and rarely diagnosed with lymphocytic esophagitis.

**Case presentation:**

Here, we present a rare case of lymphocytic esophagitis in a patient with multiple allergies and suspected eosinophilic esophagitis. A 28-year-old woman with polyvalent sensitization to food and inhalant allergens presented with intermittent dysphagia, a sensation of a foreign body in the throat, itchiness of the oral cavity after ingesting certain foods, heartburn, and prolonged chewing time. A skin prick test showed positive results for birch-tree, alder, hazel, and rye pollen, as well as house dust mites. Apart from obesity (BMI 30 kg/m^2^), multiple pustules and excoriations on the skin, her physical examination was insignificant. Esophagogastroduodenoscopy (EGD) was performed revealing full-length but discrete trachealization of the esophagus. A barium swallow test showed slowing of esophageal peristalsis in the recumbent position. No esophageal pathology was observed. A histopathological analysis of mucosal samples revealed slight hyperplasia of the basal layer of the esophagus, and the stomach showed changes typical of chronic gastritis.

**Conclusions:**

In summary, this clinical case illustrates that lymphocytic esophagitis, as a newly recognized entity, should be considered in the differential diagnosis of chronic dysphagia. Additionally, when treating allergic patients, clinicians should be aware that lymphocytic esophagitis, distinct from eosinophilic esophagitis, should be considered in the diagnosis of patients with atopy and upper gastrointestinal symptoms.

## Background

Dysphagia is defined as difficulty swallowing and represents a common symptom in the general population, with a prevalence of approximately 20% [1]. Although it may be considered a red flag for malignancies, dysphagia may also be caused by benign conditions [2]. Dysphagia is classified based on oropharyngeal or esophageal causes. Esophageal conditions presenting with dysphagia include structural abnormalities, both internal and external (compression), dysmotility, and inflammatory diseases [3]. Since dysphagia has been strongly linked with allergy, when it arises in conjunction with inflammatory conditions, eosinophilic esophagitis is most commonly suspected in patients with atopy and upper gastrointestinal symptoms. In the present case, the patient suffered from polyvalent allergies (inhalant, food, and contact dermatitis) and underwent the full diagnostic process for eosinophilic esophagitis. As a result, the patient was diagnosed with lymphocytic esophagitis, a poorly understood condition that is considered to be associated with allergy.

## Case presentation

A 28-year-old woman with polyvalent sensitization to food and inhalant allergens presented with intermittent dysphagia, sensation of a foreign body in the throat, itchiness of the oral cavity after ingesting certain foods, heartburn, and prolonged chewing time.

The initial symptoms of sneezing, nose blockage, itching in the nose, and watery eyes were first noticed by the patient five years prior during the spring. There were no allergic symptoms experienced by the patient during childhood or in her family medical history. The patient’s symptoms of allergic rhinitis and conjunctivitis had increased over the past three years and occurred during the period from March to June. Over the past two years from March until June, the patient experienced a dry cough, wheezing, and shortness of breath ("feeling of a lack of air"). The patient was diagnosed with allergic asthma approximately 2 years prior. It should be emphasized that the symptoms only occurred in the spring. The spirometry results (lung ventilation parameters assessed in the baseline spirometry test (FEV1% VCmax-88%—9th percentile, FEV1-117%) and after the diastolic test (FEV1% VCmax-90%-14percentile, FEV1-122%) were normal, and there was no significant increase in FEV1 or FVCex after administering an antispasmodic.

Our patient experienced symptoms of itchy lips, as well as tongue and mouth burning after eating hazelnuts, almonds, apples, plums, and pears. We believe that these symptoms are an expression of food allergy in the form of oral allergy syndrome (OAS). No food provocation tests were performed on our patient.

While the patient does not follow any special elimination diets, she avoids the consumption of nuts, apples, plums and pears due to OAS symptoms. However, she can eat apples and plums following heat treatment without experiencing any disturbing symptoms. During the worsening of symptoms, the patient takes inhaled corticosteroids. Asthma is well controlled.

The patient also suffered from chronic, recurrent contact dermatitis of the skin following exposure to metal parts of clothing and jewelry. She reported no family history of allergic or gastroenterological diseases.

A skin prick test was performed and showed positive results for birchtree, alder, hazel, and rye pollen, as well as house dust mites. A patch test revealed positive results for nickel sulfate. sIgE revealed positive results for house dust mites (class 4) and birchtree (class 2), but was negative for all food allergens. We performed skin prick tests for food allergens and an IgE food panel on the patient’s blood serum with a standard set of allergens. While the skin tests for hazelnuts and anise were positive in the SPT the rest were negative. The sIgE showed class 1 for milk, class 2 for beef, and the remaining sIgE were negative (Table 1).

**Table 1:** Allergy diagnostic assessments: skin-prick tests, skin (prick-by-prick) tests with native allergens, and allergen-specificIgE tests

Skin-prick tests	W	F	allergen-specific IgE	Results
Hazelnut	3	0	Beef (f27)	0.98 kU/L (allergen class 2)
Anise	3	0	Milk (f020)	0.24 kU/L (allergen class 1)
Hazel	8	23	Birch pollen ((t03)	0.85 kU/L (allergen class 2)
Alder	8	30	Gray alder pollen (t04)	0.34 kU/L (allergen class 1)
Birch	15	35	Timothy meadow (g06)	0.55 kU/L (allergen class 2)
Grasses	10	25	Rye pollen (g12)	0.47 kU/L (allergen class 2)
Rye	6	22	Mugwort pollen (w06)	0.31 kU/L (allergen class 1)
Mugwort	10	29	*D. pteronyssinus* (d01) *D. farinae* (d02)	43 kU/L(allergen class 4) 6.9 kU/L (allergen class 3)
Guinea pig	4	10		
Horse hair	6	20		
Hamster hair	3	20		
*D. farinae*	18	38		
*D. pteronyssinis*	8	25		
Positive control	3	15		
Negative control	0	0		

W: wheal; F: flare

Apart from obesity (BMI 30 kg/m^2^), multiple pustules and excoriations on the skin, the physical examination was insignificant. Laboratory findings, including a complete blood count, liver and kidney function tests, and coagulogram were unremarkable.

**Fig. 1:**
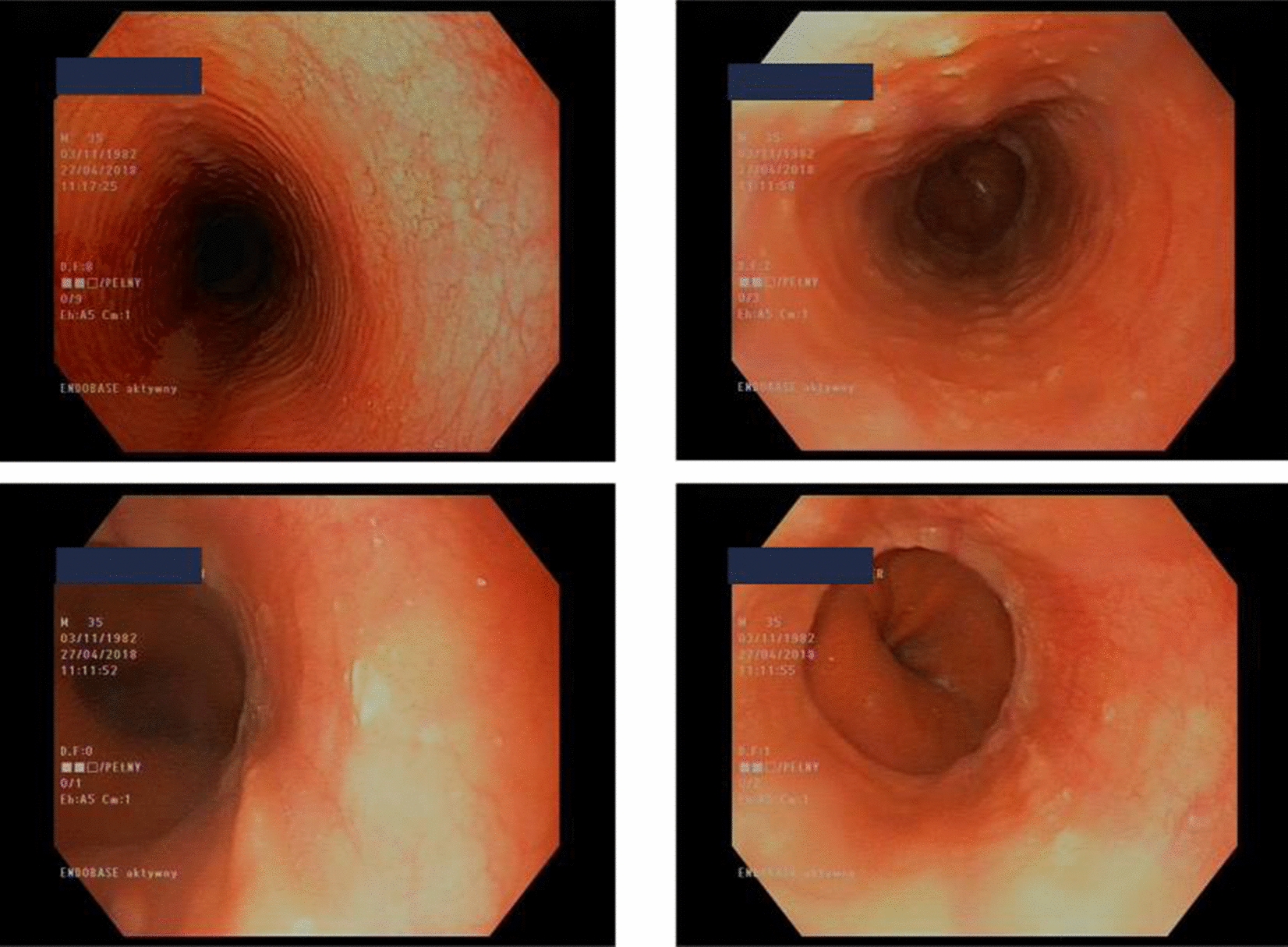
Full-length but discrete trachealization of the esophagus. (Courtesy of Department of Gastroenterology and Internal Medicine, Medical University of Warsaw)

**Fig. 2:**
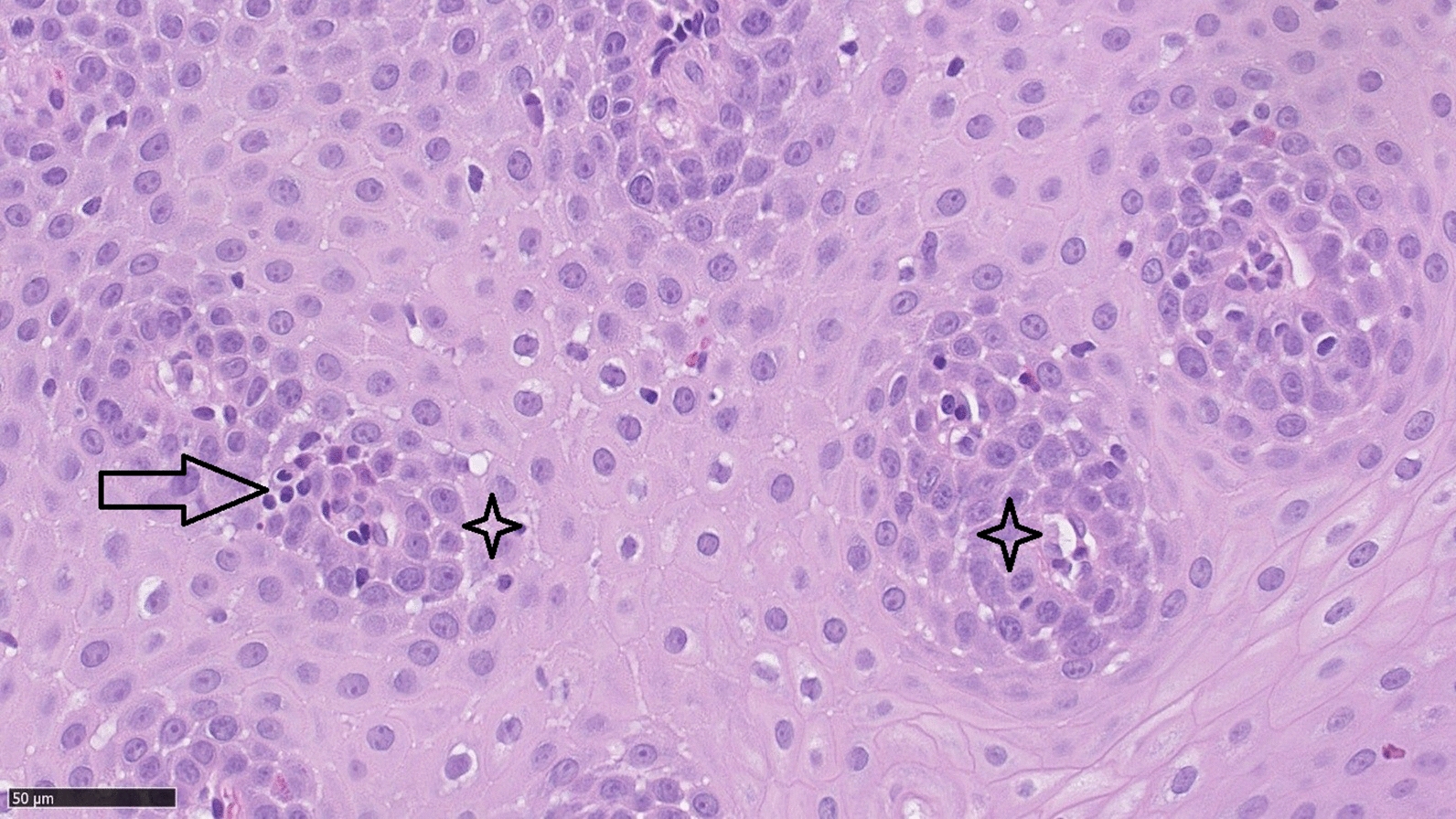
Multilayer flat epithelium with acanthosis and lymphocytic infiltrations (Courtesy of Department of Pathology, Medical University of Warsaw). Arrow: Peripapillary fields intraepithelial lymphocytes; Star: Peripapillary fields spongiosis

An esophagogastroduodenoscopy (EGD) revealed full-length but discrete trachealization of the esophagus (Fig. 1). Multiple biopsies were obtained from the proximal, middle, and distal esophagus, each of which showed multilayer flat epithelium with acanthosis and lymphocytic infiltration (Fig. 2). The histopathological examination of our patient revealed: (1) lymphocytes: up to 60/1DPW (60 cells per 1 high power field); (2) eosinophils: up to 7/1DPW, but in most fields of view up to 3–4/1DPW; and (3) neutrophils: up to 2/1DPW, many fields without neutrophils. The finding of 60 lymphocytes in the field of view and up to 7 eosinophils in the field of view appeared to confirm a diagnosis of lymphocytic esophagitis and exclude eosinophilic esophagitis (EoE).

A barium swallow test showed slowing of esophageal peristalsis in the recumbent position. Peristaltic movement was also slowed in the duodenum and small intestine, and was absent in the stomach.

A therapeutic trial of the proton pump inhibitor (PPI), pantoprazole, was initiated at a standard dose followed by an EGD and esophageal biopsy. The patient received continued care in the allergology outpatient clinic.

Before the PPI therapeutic trial had ended, the patient presented in the Emergency Room with postprandial nausea, vomiting, epigastric pain, and involuntary weight loss (5 kg over 6–7 weeks). Apart from elevated alanine transferase activity (77 µ/L), the laboratory findings were insignificant and the abdominal ultrasound was normal. In response, the following treatment modifications were introduced: the PPI dose was doubled, and both itopride hydrochloride and famotidine were initiated with no improvement. Three days later, the patient was admitted to the Department of Gastroenterology and Internal Medicine. The EGD was repeated, revealing erosions of the cardiac mucous; however, no esophageal pathologies were observed. Histopathological analysis of mucosal samples obtained from the esophagus revealed slight hyperplasia of the basal layer and those from the stomach showed changes typical of chronic gastritis. Treatment with intravenous PPI therapy for three days followed by 4 weeks of an oral therapeutic dose of pantoprazole resulted in significant improvement.

## Discussion

Although lymphocytic esophagitis (LyE, LE) was first described in 2006, the diagnostic criteria and pathogenesis remain unclear. In 2006, Rubio et al. described 20 patients exhibiting increased intraepithelial lymphocytes with few intraepithelial granulocytes in the peripapillary fields of esophageal mucosal samples [4]. Based on different reported cases of LyE, a wide-range of lymphocyte numbers have been proposed as a threshold for diagnosis [5]. A minimum of 20 lymphocytes per high power field was first proposed by Rubio et al. [4]. The study conducted by Haque et al. [6] described LyE as dense lymphocytic infiltration of the peripapillary squamous mucosa (the number of lymphocytes was not defined) with marked spongiosis and few neutrophils and eosinophils. The authors concluded that defining the threshold number of lymphocytes would be misleading and lower the specificity of the diagnosis. Both Rubio et al. and Purdy et al. agree that the above described changes appear only in the esophageal mucosa in LyE. Moreover, specimens acquired from different parts of the gastrointestinal tract, including the stomach, small intestine, and colon are normal in patients with LyE [4, 7].

Demographic studies have shown that LyE is identified in 1 of every 1000 patients. In the study performed by Genta et al., LyE was identified in only 116 out 129,525 biopsies [8].

The clinical manifestation of lymphocytic esophagitis is inconsistent between different reports. Most commonly reported symptoms include dysphagia for solids, chest discomfort, heartburn, and food impaction [9–11]. However, one of the first reports by Purdy et al. compared 42 patients with LyE to 34 control individuals and found no differences in symptoms [7].

While the pathogenesis of lymphocytic esophagitis remains unclear, associations between LyE and different conditions have been proposed. T cells are involved in the induction of allergen sensitization, the activation of this process, and in the control of allergic inflammation. T cells drive the immune response and can be divided into two groups depending on the structure of the TCR receptor: (1) α/β receptor expressed by helper Th, cytotoxic Tc, and NK (LGL) cells; and (2) γ/δ receptor expressed by cells located in the lymphoid organs. TCR receptors each have different specificity, and Th1 cell profiles support a cellular immune response, whereas the Th2 population plays a role in the modulation of allergic inflammation. Thus, T cells appear to be the most important cells in both allergic and lymphocytic esophagitis [12].

An individual who comes into contact with an allergen responds by producing specific IgM, IgG, or IgE antibodies and generates specific lymphocyte clones that recognize these antigens. The consequence of the first contact with an allergen is sensitization, the process of activating antigen-specific T cells or plasma B cells that produce antigen-specific antibodies. Upon subsequent contact with the allergen, an inflammatory reaction is induced, termed an allergic reaction. If this reaction is clinical, it is termed an allergic disease. According to the Gell and Coombs classification, type I reactions or an anaphylactic type reaction (rapid, type I hypersensitivity reaction), and type IV reactions (delayed type hypersensitivity, DTH) represent the most important forms of allergic disease pathogenesis. The diseases associated with a type I hypersensitivity reaction mechanism include urticaria, anaphylaxis, asthma, allergic rhinitis, allergic conjunctivitis, food allergy, and allergies to insect venoms. In type I hypersensitivity reactions, low concentrations of the allergen (via inhalation or exposure in the gastrointestinal tract) presented in the presence of interleukin (IL)-4, IL-5, IL-9, and IL-13 are responsible for the dominant Th2 response. In most cases, the Th2 response is associated with a genetic determinant of a dominant Th2 response, an imbalanced Th2 response preserved during pregnancy via exposure to agonists of bacterial toll-like receptor (TLR)agonists (e.g., LPS, dsRNA, and DNA CpG),or low regulatory T lymphocyte (Treg) activity. Genetics factors increase the risk of developing an allergic disease and the local environment influences gene expression, thereby modifying allergic inflammation. Undoubtedly, the mechanisms of epigenetic regulation play an important role in the development of allergic diseases, the most important of which are DNA methylation, modification of histone proteins, and non-coding RNA molecules. In a type I hypersensitivity, contact with the antigen(allergen) in a Th2-polarized environment leads to the production of IgE, which binds to the FceR1 receptor expressed on the surface of mast cells and basophils. The binding of the allergen to IgE leads to mast cell activation and subsequent degranulation. In contrast, a type IV hypersensitivity involves only cellular response mechanisms, which consist of both CD4 and CD8 T cells. The antigen is presented by dendritic cells to Th1 cells, which activate macrophages and cytotoxic T lymphocytes (Tc). The essential cytokines that mediate inflammation in a type IV reaction are IL-1β, TNF-α, and GM-CSF. These cytokines are typically responsible for the inflammatory cell infiltration observed in these reactions, which usually reaches its peak intensity 24–48 h after antigen exposure. In a type IV hypersensitivity reaction, the dominant activity of CD8 lymphocytes in the effector phase represents the most important pathogenetic element in eczema. Although the pathogenic mechanism of lymphocytic esophagitis is currently unknown, Purdy et al. believe that the mechanism is similar to that of contact eczema (i.e., a type IV hypersensitivity reaction with a dominance of T cells in the effector phase). The involvement of lymphocytes in the pathogenesis of allergic diseases and lymphocytic esophagitis may indicate a link between these diseases; however, further research is required to confirm this hypothesis. Interestingly, in our patient, we also observed the coexistence of allergic skin eczema in the context of a nickel allergy and lymphocytic esophagitis, in addition to allergic rhinitis and asthma [12–16].

Nevertheless, published reports are not consistent. Habbal et al. conducted a systematic review of 14 studies involving 379 patients and found that the most common associations were motility disorders (12.0%), smoking tobacco (12.7%), alcohol consumption (11.4%), hypothyroidism (9.4%), allergies (7.4%), and asthma (5.4%) [12]. In the study conducted by Purdy et al., allergies were reported in 33% of patients with LyE. Allergic conditions in these patients included seasonal allergy, asthma, and celiac disease. While one patient was diagnosed with a food allergy, it is likely that some patients had unrecognized food allergies or nonallergic reactions to ingested substances [7]. Although the authors did not find significant differences between the LyE and control patients regarding the prevalence of allergies and any other specific diseases, they concluded that lymphocytic esophagitis resembled contact dermatitis. Since our patient was previously diagnosed with asthma, contact dermatitis, and seasonal allergies, it is possible that one of these allergic conditions may be the underlying cause of the observed lymphocytic inflammation.

Histologically, lymphocytic esophagitis is characterized by spongiosis and high numbers of intraepithelial lymphocytes located primarily around peripapillary fields, with a complete absence or minimal number of granulocytes. The histopathological findings reported in the original study by Rubio et al. included the expression CD3 and CD4 proteins by 42% of the intraepithelial (IEL) lymphocytes, and 36% of the IELs expressed CD8. Moreover, granzyme B was expressed by 0.2% and T cell intra cytoplasmatic antigen (TIA) 1 was not expressed [4]. The study conducted by Xue et al. analyzed lymphocyte subsets and found that primary motility disorders occurred more frequently in patients with CD4 predominant esophagitis than in those dominated by CD8 lymphocytes [9].

Cohen et al. reported that gastrointestinal reflux disease was the most common association (49%) in as tudy of 81 patients with LyE. In first report of the entity association between Crohn’s disease (CD) had been proposed. In the original study by Rubio et al., 40% of the cohort suffered from CD. This finding was followed by studies in both pediatric and adult populations, which found no association in adults [11]. In contrast, in children, the prevalence of LyE in patients with CD varies between 12 and 28%, depending on the study [13, 14].

Endoscopically, LyE may mimic EoE, including the presence of linear furrows, whitish exudates, strictures, or stenosis [6]. Moreover, normal esophageal mucosa is observed in up to 55% of cases of LyE [6]. Tanaka et al. attempted to define the differences between the endoscopic features of LyE, EoE, and GERD using narrow band imaging magnifying endoscopy (NBI-ME). The studied features included beige colored mucosa, invisible submucosal vessels, as well as an increased and dot-shaped congested intra papillary capillary loop. In a group of LyE patients, 91% presented with at least one of these three features, and all three were observed in 82%. In the EoE cohort, all three features were observed in every patient. In the GERD cohort, none of the patients presented all three features and only one third presented with at least one [17]. Since there are no formal guidelines for the treatment of LyE, management of the condition can be challenging. Symptomatic management is similar to the treatment for EoE, which includes PPIs, swallowing topical steroids (e.g., fluticasone), and endoscopic dilatation in severe cases of narrowing [17, 18]. The management of LyE has not been addressed in many studies to date, likely due to the low prevalence of this disease.

Cohen et al. found LyE to be a relatively benign disease, as 59% of their cohort study improved in response to PPI therapy with no significant long-term complications. The highest rate of improvement was observed in patients with dysphagia and the lowest in those with chest pain. Moreover, 9 of the 22 patients continued to exhibit LyE changes in the follow-up biopsy [11]. One case of spontaneous esophageal perforation was reported in a patient with LyE; however, the association is unclear [19].

Unfortunately, LyE remains a poorly understood disease and there is a lack of information regarding the use of biologically derived drugs for this disease in the published literature. In addition, most publications are associated with gastroenterological research. However, the findings from our patient highlights the necessity of multidisciplinary care for patients with this disease.

## Conclusions

Further studies clarifying the pathogenesis, clinical manifestation and progression and most importantly, the course of treatment are required to gain a greater understanding of LyE. In summary, we present this clinical case to illustrate that LyE, as a newly recognized entity, should be considered in the differential diagnosis of chronic dysphagia. In addition, the role of allergies in the pathomechanism of LyE remains unclear. The goal of this case report is to draw the attention of allergists to this clinical problem, demonstrating that both EoE and LyE can be suspected in patients presenting with allergies and upper gastrointestinal symptoms. Since the diagnosis of this disease is based on the results of an endoscopic examination and esophageal biopsy, cooperation with gastroenterologists and pathomorphologists in this field is necessary.

## Data Availability

All data generated or analysed during this study are included in this published article.
